# Natural Layered Phlogopite Dielectric for Ultrathin Two-Dimensional Optoelectronics

**DOI:** 10.1021/acsnano.5c09046

**Published:** 2025-08-08

**Authors:** Thomas Pucher, Julia Hernandez-Ruiz, Guillermo Tajuelo-Castilla, José Ángel Martín-Gago, Carmen Munuera, Andres Castellanos-Gomez

**Affiliations:** † 2D Foundry Research Group. 69570 Instituto de Ciencia de Materiales de Madrid (ICMM-CSIC), Madrid E-28049, Spain; ‡ ESISNA Research Group. 69570 Instituto de Ciencia de Materiales de Madrid (ICMM-CSIC), Madrid E-28049, Spain

**Keywords:** 2D materials, TMD, MoS_2_, mica, dielectric, high-k, phototransistor

## Abstract

The integration of high-dielectric-constant (high-κ) materials with two-dimensional (2D) semiconductors is promising to overcome performance limitations and reach their full theoretical potential. Here, we show that naturally occurring phlogopite mica, exfoliated into ultrathin flakes, can serve as a robust high-κ dielectric layer for transition metal dichalcogenide-based 2D electronics and optoelectronics. The wide band gap (∼4.8 eV), high dielectric constant (∼11), and large breakdown field (>10 MV cm^–1^) of phlogopite enable transistors with subthreshold swings down to 100 mV dec^–1^, minimal hysteresis (∼30–60 mV), and interface trap densities comparable to those of state-of-the-art oxide dielectrics. Moreover, phototransistors built upon monolayer molybdenum disulfide (MoS_2_) and phlogopite exhibit responsivities up to 3.3 × 10^4^ AW^–1^ and detectivities close to 10^10^ Jones, surpassing devices based on conventional gate insulators. We further demonstrate the versatility of this natural dielectric by integrating phlogopite/MoS_2_ heterostructures into NMOS inverters, showcasing robust voltage gains and low-voltage operation. Our findings establish phlogopite as a promising, earth-abundant dielectric for next-generation 2D transistor technologies and high-performance photodetection.

## Graphical abstract

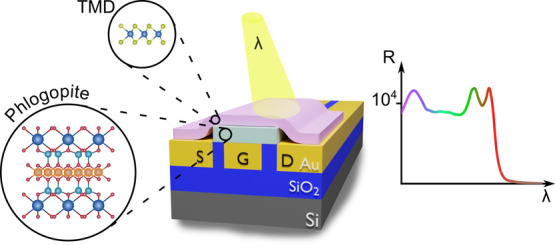

Two-dimensional (2D) materials have attracted considerable attention in electronics and optoelectronics due to their atomic thinness and outstanding properties such as high carrier mobility and tunable band gaps.^1,2^ Among these, transition metal dichalcogenides (TMDs), particularly MoS_2_, have shown remarkable potential in ultrathin transistor and photodetector applications.^3–10^ Nonetheless, achieving optimal performance in 2D electronic devices requires integration with suitable gate dielectrics characterized by high dielectric constants (κ), low leakage currents, large dielectric breakdown strength, and minimal interfacial defects.^11^

Traditionally employed high-κ oxides such as hafnium oxide (HfO_2_) or aluminum oxide (Al_2_O_3_) suffer from inherent limitations such as amorphous structure-related defects, surface roughness, and deposition-induced damage to the sensitive surfaces of TMDs. Such drawbacks significantly degrade the carrier mobility, introduce hysteresis, and diminish the subthreshold swing, critically restricting the device efficiency and reliability. Recently, crystalline dielectrics, such as SrTiO_3_,^12,13^ BaTiO_3_,^14^ Bi_2_SeO_5_,^15,16^ Sb_2_O_3_,^17,18^ or MgNb_2_O_6_,^19^ have been explored to overcome these challenges. Despite their advanced electrical performance improvements, these studies fail to acknowledge the optoelectronic promises of 2D semiconductors, by simply not considering to characterize the photoresponse of their devices and focusing only on electronic metrics. Perovskite oxides, such as Sr_2_Nb_3_O_10_, have been proposed to achieve dual functionalities of dielectric gating and photodetection.^20^ However, these materials present challenges in terms of the ease of fabrication and assembly and limited achievable responsivities compared to other heterostructures. Nur et al. highlight the crucial dual role of the dielectric in 2D phototransistors:^21^ providing a high dielectric constant and hosting charge traps that govern photogating gain. First, the interface between the dielectric and semiconductor directly controls the density and lifetime of traps; a clean, atomically flat interface enables controlled, reproducible photogating, while uncontrolled traps can increase noise or slow device response. Second, a high-κ dielectric is essential not only for strong gate coupling but also for screening Coulomb scattering, which boosts mobility and can enhance light–matter interactions. Importantly, the photogating gain mechanism depends on the ratio of trap lifetime to carrier drift time.^22^ Thus, to achieve a high gain without a slow response, the system requires both efficient trapping and high mobility. These considerations show that ideal dielectrics for 2D optoelectronics must offer controlled interfaces for trap engineering, high κ for mobility and electrostatics, and optical transparency.

Although naturally occurring layered dielectrics, such as micas, have been proposed to overcome fabrication complexities and achieve high-performing optoelectronic 2D devices,^23–26^ so far, such results have not been reported.

Here, we introduce naturally occurring layered phlogopite mica as a highly promising alternative dielectric that addresses many of these limitations. Phlogopite mica, mechanically exfoliated from bulk crystals, exhibits exceptional dielectric characteristics, including a high dielectric constant (∼11), robust dielectric breakdown fields (>10 MV cm^–1^), and a wide optical band gap (4.8 eV). Our devices, integrating phlogopite with MoS_2_, demonstrate outstanding electrical performance with significantly reduced hysteresis, steep subthreshold swings (as low as 100 mV dec^–1^), and minimal interfacial trap densities. Our phlogopite-based MoS_2_ phototransistors achieve extraordinary optoelectronic performance, exhibiting responsivities reaching 3.3 × 10^4^ AW^–1^, photogain up to 6 × 10^4^, and high detectivities (∼10^10^ Jones). These metrics highlight the intrinsic advantage of phlogopite in optoelectronic applications and its potential to advance high-performance, multifunctional 2D electronics and photonics.

## Results and Discussion

We begin by analyzing the material properties of our phlogopite crystal. A representation of the atomic structure of a single phlogopite layer is shown in Figure 1a (crystal structure adapted from ref 27). Phlogopite (KMg_3_(AlSi_3_)­O_10_(OH)_2_) belongs to the mica family, a group of earth-abundant minerals and subset of phyllosilicates commonly found in the Earth’s crust. Its structure is predominantly composed of oxygen, silicon, and aluminum, and it crystallizes in a monoclinic system. The material adopts a layered sheet-like morphology due to its characteristic tetrahedral–octahedral–tetrahedral (T–O–T) stacking structure.^28–30^ In this 2:1 phyllosilicate framework, an octahedral sheet of cations is sandwiched between two sheets of linked (Si, Al)­O_4_ tetrahedra.^31^ Compared to muscovite, another commonly studied mica, phlogopite is richer in magnesium and poorer in aluminum. Its color can vary from golden brown (see Figure 1a, inset) to reddish-brown depending on the presence of impurities such as iron (Fe), titanium (Ti), or fluorine (F), which can substitute for hydroxyl (OH^–^) groups. Increasing the Fe content causes a transition toward biotite, while F enhances the thermal stability.^32^ The TOT structure promotes easy cleavage along the basal planes, where the mesh of basal oxygen atoms facilitates mechanical exfoliation of flakes with thicknesses ranging down to the ultrathin limit (Figure 1b).^33^

**Figure 1:**
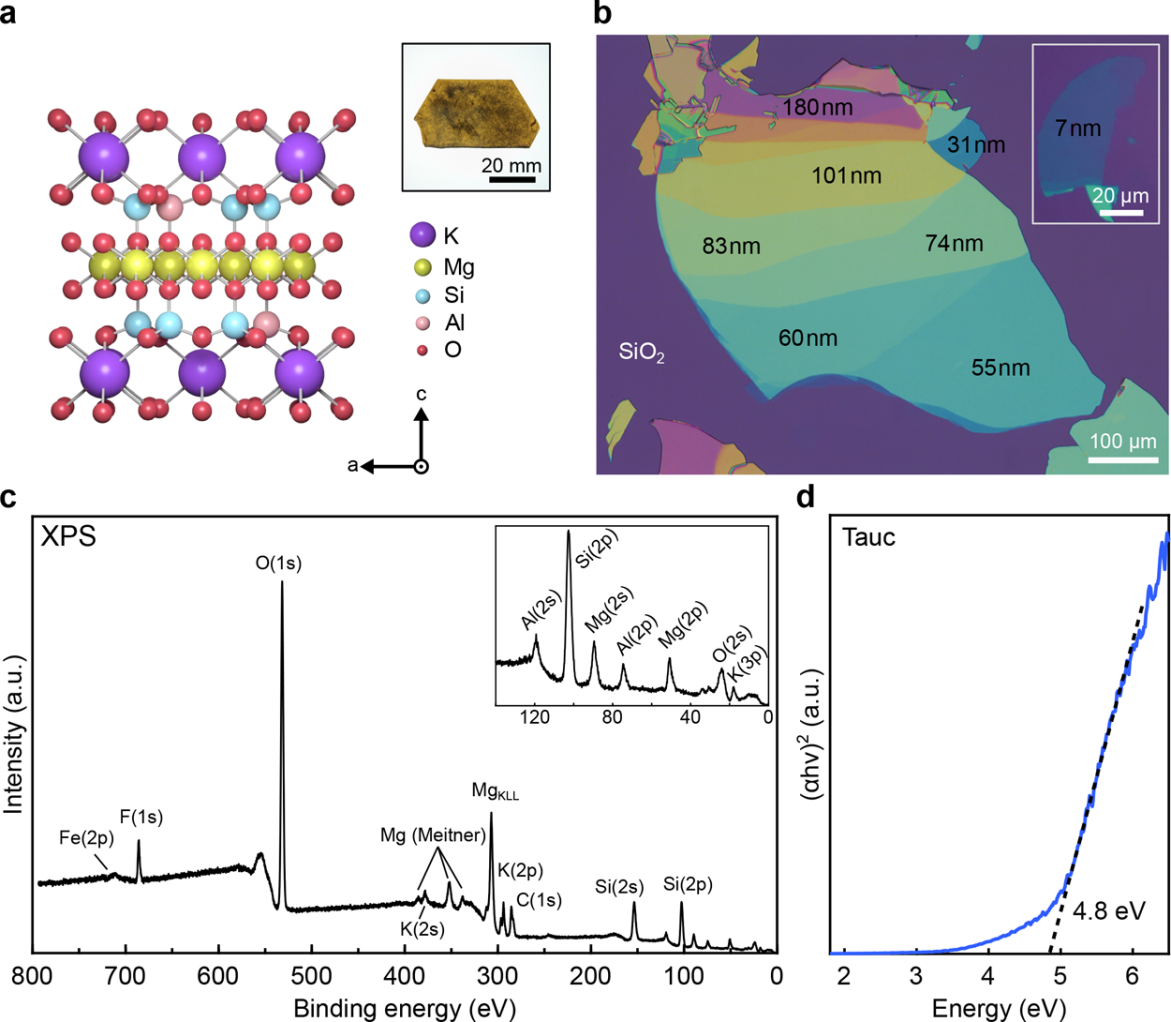
Phlogopite material properties. (a) Atomic structure of one-layer phlogopite including crystal axes. (Inset: optical image of the bulk crystal showing brown color). (b) Optical micrograph of layered phlogopite flakes, illustrating the rich optical contrast of different layer thicknesses down to 7 nm (inset) on a 290 nm SiO_2_/Si substrate. (c) XPS spectrum of the phlogopite crystal. Mg and K represent the most prominent peaks. An almost diminishing iron peak Fe­(2p) proves that our crystal shows low impurity content. (d) UV–vis absorption spectroscopy of thin phlogopite layers represented by a Tauc plot of direct optical transition (α = 2), revealing an optical band gap of 4.8 eV.

We employ the conventional Scotch-tape method to exfoliate phlogopite onto PDMS (Gel-Pak) substrates, followed by a standard dry-transfer technique^34^ to relocate the flakes onto desired substrates. The weak van der Waals forces between the layers facilitate mechanical exfoliation of flakes down to the monolayer limit.^24,35,36^ Flake thicknesses can be easily distinguished by color appearance, resulting from differences in the optical contrast on SiO_2_ substrates.^37–39^ Using microreflectance spectroscopy and applying Fresnel law equations, we can determine the layer thickness with high accuracy directly on the substrate, as previously demonstrated for other mica materials.^40^ Out of these measurements, we can also determine the refractive index for phlogopite, yielding 1.45, consistent with earlier reports.^40^ To verify the accuracy of the optical contrast thickness determination, we measured the topography of the flake in Figure 1b by atomic force microscopy (AFM, Supporting Information Figure S1). To assess the elemental composition, we performed X-ray photoelectron spectroscopy (XPS) on exfoliated phlogopite flakes transferred onto SiO_2_ (290 nm)/Si substrates. The resulting spectrum (Figure 1c) confirms Mg and K as the dominant substituents and shows negligible Fe­(2p) and no detectable Ti peaks, indicating a very low impurity content. Trace amounts of fluorine (F 1s) are detected and are expected to enhance the thermal stability. The XPS results confirm that the elemental composition of phlogopite includes major constituents such as silicon (44.2%) and oxygen (33.4%) also present in the substrate, minor constituents such as magnesium (8.9%), aluminum (8.8%), and potassium (2.4%), and trace amounts of iron (0.7%) and fluorine (1.7%). Raman spectroscopy of bulk phlogopite (see Supporting Information) yields vibrational modes in agreement with the literature,^41,42^ further validating the crystal quality. To determine the optical band gap, we performed UV–vis absorption spectroscopy on thin exfoliated phlogopite flakes (Figure 1d). Plotting the data as a Tauc plot with a direct optical transition^43^ reveals a band gap of 4.8 eV. This value is slightly higher than those previously reported for natural phlogopite (3.6 eV)^24^ and lower than that of synthetic fluorphlogopite (∼7.8 eV).^44^ The difference likely stems from the low Fe impurity content of our sample, as discussed in earlier studies.^24,44^ Studies on other dielectric materials have shown the effect of Fe or F on their dielectric properties. Fe impurities in lanthanum oxide (La_2_O_3_), for example, result in a decrease in its dielectric constant,^45^ whereas fluorination of hexagonal boron nitride (hBN) is the reason for a bandgap reduction and a lower dielectric constant.^46,47^ Notably, many high-κ dielectrics suffer from relatively low band gaps (<4 eV), which reduce the tunneling barrier and lead to higher leakage currents.^14,48,49^ The measured band gap of 4.8 eV of our natural phlogopite, therefore, provides a favorable combination of low leakage and high dielectric strength, making it an attractive candidate for use in 2D optoelectronic devices. Phlogopite, or more critically its synthetic version fluorphlogopite, can also be a promising dielectric material for large-scale applications in combination with CVD-grown semiconductors. Recent studies have shown that fluorphlogopite can serve as a substrate to epitaxially grow single-crystal dielectrics, such as In_2_Se_3_ or MgNb_2_O_6_, and how to combine them with CVD-grown 2D semiconductors.^19,50^

### Dielectric Properties of Thin Phlogopite Flakes

Dielectric strength and insulating properties are critical in 2D optoelectronic applications where selecting an appropriate dielectric layer can substantially influence the device performance. Here, we use three complementary methods to characterize the dielectric constant of thin phlogopite flakes: conductive AFM (C-AFM), double-gated field-effect transistor (FET) measurements, and traditional metal–insulator–metal (MIM) capacitance tests. Although MIM capacitance measurements can yield accurate results for sufficiently large and homogeneous flakes, fabricating large-area parallel-plate structures from ultrathin phlogopite is often challenging. Hence, we primarily rely on C-AFM and double-gated FET approaches for thinner specimens.

First, C-AFM enables point-by-point measurement of tunneling current through phlogopite, allowing multiple measurements at different locations on the same flake. Additionally, AFM provides information about surface roughness and contaminants. As shown in Figure 2a–c, a 6.8 nm flake transferred onto a platinum (Pt) electrode is scanned with a platinum AFM tip, where local current–voltage (*I*–*V*) sweeps are performed at multiple spots on the same flake. The resulting *I*–*V* curves can be fitted by a Schottky emission model, which directly relates the slope of the tunneling current to the dielectric constant^51,52^ (Figure 2c and more details on the model and fit in the Supporting Information). From 13 measurement spots, we obtain an average dielectric constant of 10.7. Simultaneously, this approach provides insights into the dielectric strength of phlogopite by monitoring *I*–*V* sweeps until breakdown, yielding a breakdown field of ∼11.3 MV cm^–1^. This value is comparable to those reported for other micas^23,25^ and significantly surpasses those of newly proposed dielectric materials for 2D electronics.^12,53,54^ Moreover, it exceeds the 1 V nm^–1^ minimum breakdown field set by the International Roadmap for Devices and Systems (IRDS).^55^ Breakdown measurements on flakes of 6.8 and 22 nm thickness and more details on extracting the dielectric constant from the *I*–*V* slopes are presented in the Supporting Information.

**Figure 2:**
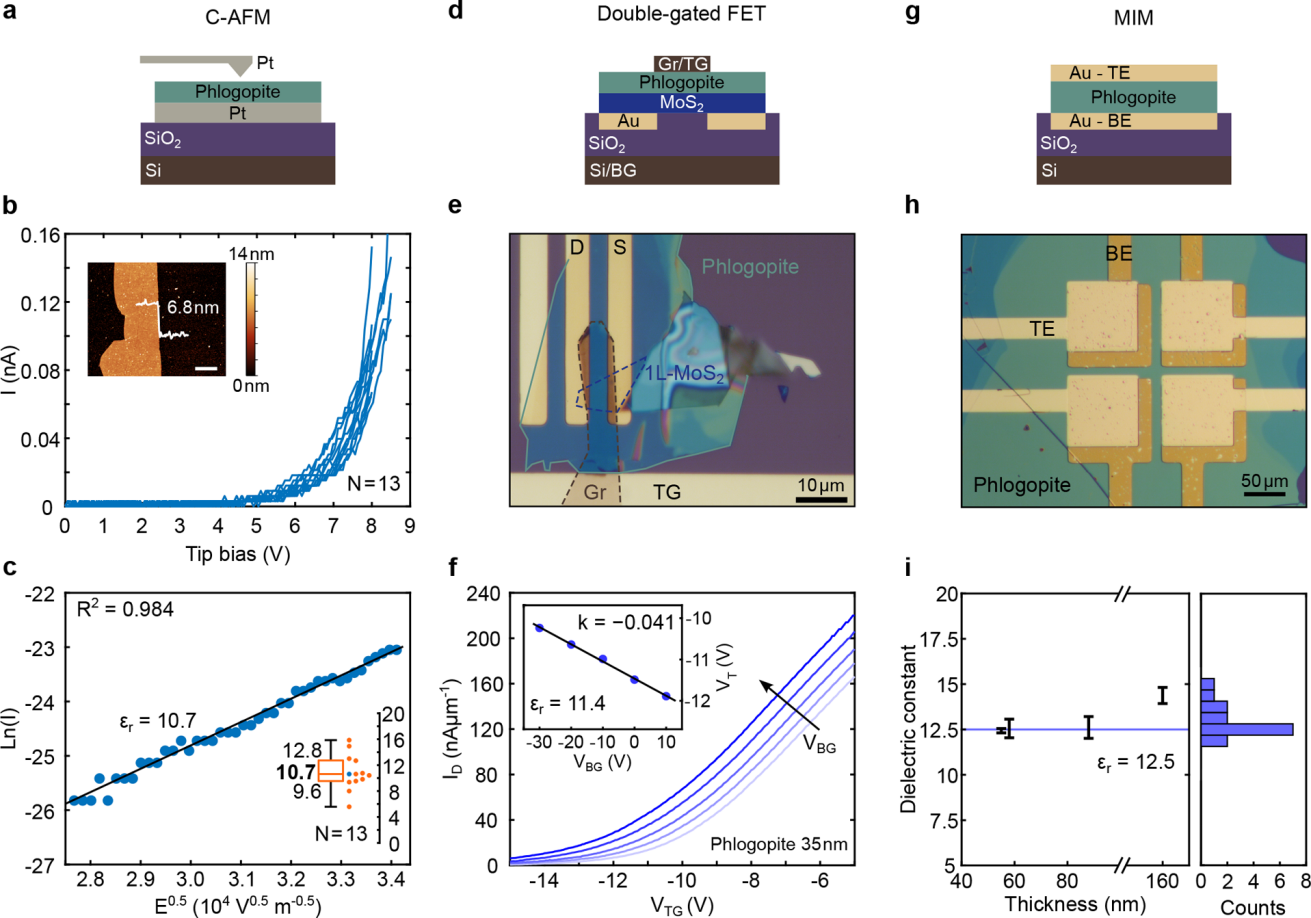
Dielectric properties of layered phlogopite by three different methods. (a–c) Determination of ultrathin layers of phlogopite by C-AFM. Schottky emission of a flake with a thickness of 6.8 nm was measured at multiple spots of the flake (b). The scale bar of the inset is 2 μm. All curves were fitted to the Schottky emission model, and a mean dielectric constant of 10.7 was extracted (c). (d–f) Top- and bottom-gated Gr/Phlogopite/MoS_2_/SiO_2_ heterostructure for dielectric constant extraction (*W* = 5 μm, *L* = 3 μm). The threshold voltage (*V*_T_) shift of the transistor is recorded by sweeping the top-gate voltage for different back-gate biases (f). Extracting the slope k under a linear fit (f, inset) gives the dielectric constant by relating the top (phlogopite) and bottom capacitances (SiO_2_). (g–i) MIM capacitance measurements. Prepatterned buried gold pads serve as the bottom electrode (BE), where large phlogopite flakes are transferred on top. Top gold electrodes (TEs) are patterned using a maskless photolithography process to form an MIM structure. Capacitances are evaluated for four different thicknesses at *f* = 100 Hz, with four devices for each thickness. Dielectric constants for these thicknesses (extracted using the parallel-plate capacitor model) are shown in (i).

Next, we employed double-gated FET structures to validate these findings. A monolayer MoS_2_ FET is fabricated on an SiO_2_(290 nm)/Si substrate, then capped with a 35 nm phlogopite flake, and at last top-gated with a graphite flake (Figure 2e). By recording the shift of the top-gate threshold voltage (*V*_T_) under different back-gate biases, and modeling both gates as parallel-plate capacitors, we can extract the dielectric constant of phlogopite from the slope of the shift of *V*_T_ (inset of Figure 2f), by using the following relationship^15,56,57^

$$\frac{C_{{\mathrm{SiO}}_{2}}}{C_{P}}=\frac{\varepsilon_{{\mathrm{SiO}}_{2}}\times t_{P}}{\varepsilon_{P}\times t_{{\mathrm{SiO}}_{2}}}$$ 1

where ε_SiO_2__ and ε_P_ are the dielectric constants of SiO_2_ (3.9) and phlogopite, respectively, and *t*_SiO_2__ and *t*_P_ are their respective thicknesses. Specifically, the ratio of the top-gate to bottom-gate capacitance leads to a dielectric constant of 11.4 (Figure 2f), which closely agrees with the C-AFM-based measurements.

Lastly, we conduct conventional MIM capacitance experiments for thicker flakes (50 nm and above), where uniform large-area flakes are easier to achieve (Figure 2g–i). We transfer 500 × 500 μm flakes onto prepatterned Au electrodes and then pattern and evaporate top Au contacts via maskless lithography to form parallel-plate capacitors. Measuring the capacitance at 100 Hz for four different flake thicknesses up to 160 nm results in an average dielectric constant of 12.5 (Figure 2i). Measurements at 1 kHz are included in the Supporting Information. Overall, dielectric constants of 10.7–12.5 place phlogopite well above hBN (∼3)^58^ and at par with well-established materials such as Si_3_N_4_ or Al_2_O_3_.^48^ Together with the electric breakdown field, we compare phlogopite with other proposed dielectric materials for 2D electronics in the Supporting Information (Figure S5).

### Natural Phlogopite as an Insulator for 2D Electronics

To fabricate 2D phototransistors, we integrate mechanically exfoliated monolayer MoS_2_ with phlogopite as a gate insulator. We employ buried bottom-gated electrodes on SiO_2_(290 nm)/Si­(p^+2^) wafers, as illustrated by the 3D schematic in the inset of Figure 3a. The electrodes are patterned via maskless lithography and formed by a double-layer resist process to create undercut resist profiles. Applying a glass-etching cream to the exposed regions of SiO_2_ produces 50 nm trenches, into which 5 nm Cr/45 nm Au is thermally evaporated, resulting in a planar surface between the electrodes and the SiO_2_ substrate (see ref 59 for more fabrication details). This approach yields prepatterned substrates optimized for transferring 2D materials while minimizing mechanical stress.

**Figure 3:**
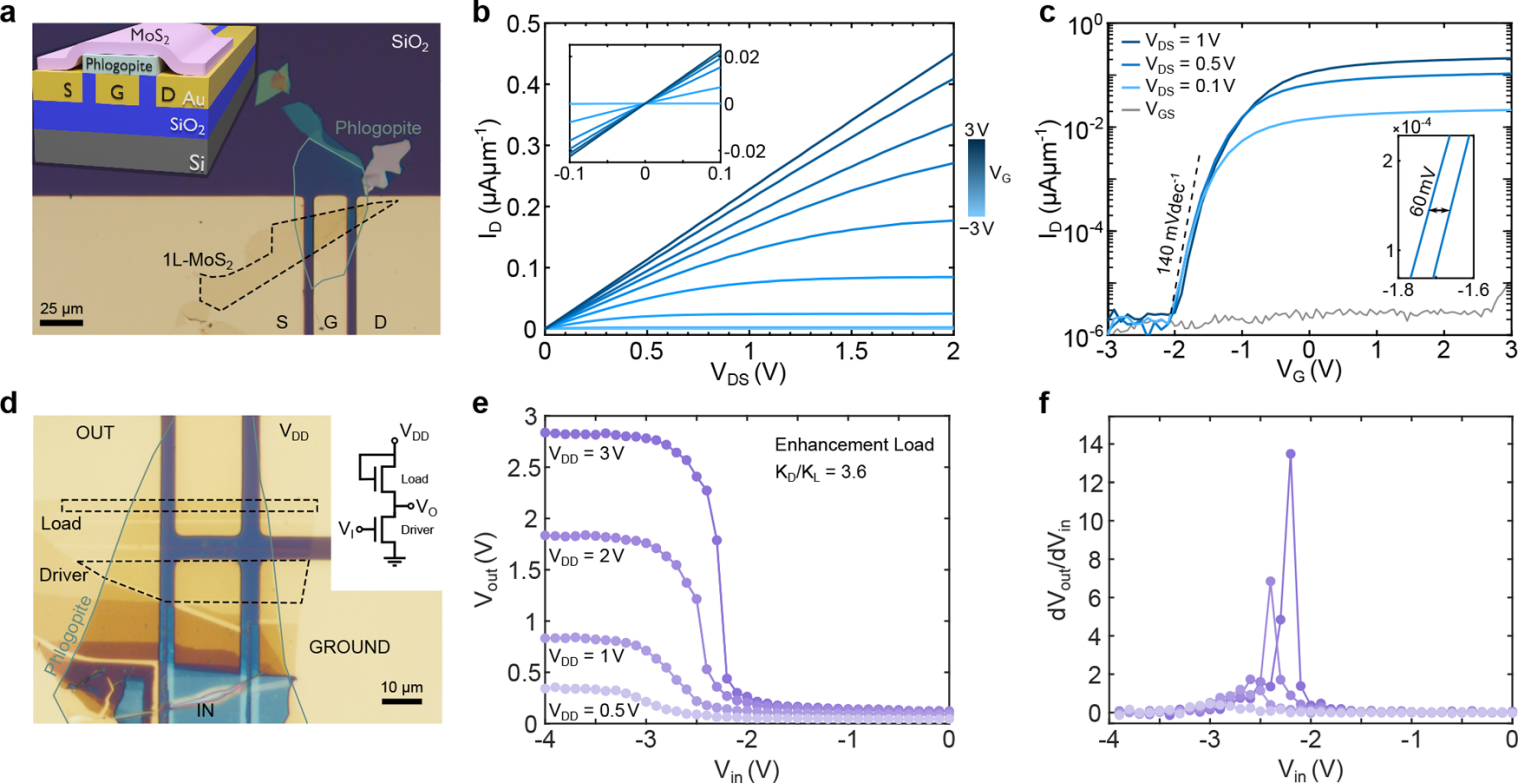
Monolayer MoS_2_ transistor and digital circuit inverter with phlogopite dielectric. (a) Optical micrograph of single-layer MoS_2_ transistor on buried gold electrodes with a 11 nm thick phlogopite (*W* = 14 μm, *L* = 25 μm). The inset shows a 3D sketch of the device architecture. (b) Gate-dependent source–drain current–voltage (*I*–*V*) curves showing current saturation at a higher *V*_DS_ and linear behavior at low *V*_DS_ (inset). (c) FET forward transfer characteristics for different bias voltages and gate leakage. The inset shows the hysteresis of forward vs backward sweeps for *V*_DS_ = 0.5 V. (d) Optical micrograph of a single-layer MoS_2_ NMOS inverter structure with enhancement load. The inset illustrates the analog circuit design. All electrodes are again buried and therefore planar with the SiO_2_ surface. Both transistors use the same phlogopite and MoS_2_ flake by design. The MoS_2_ was laser-patterned with a 532 nm laser of a Raman system to define an enhancement load ratio of around 4 (ratio between the widths of the two transistors, *L* = 25 μm). (e) Inverter characteristics for different V_DD_ voltages. A small voltage drop in comparison to *V*_DD_ is natural due to the enhancement load of the circuit. (f) Inverter gain extracted from (e).

As the next step, an 11 nm thick flake of phlogopite is transferred onto the substrate to isolate the central gate electrode (*G*). A monolayer of MoS_2_ is then transferred to span the gap between the source (S) and the drain (D) contacts, as depicted in Figure 3a. Figure 3b shows the gate-dependent current–voltage (*I*–*V*) characteristics for V_DS_ up to 2 V, which exhibit current saturation. At low *V*_DS_ (inset), the linear behavior suggests low Schottky barriers.^60^ Forward transfer characteristics of the FET for different *V*_DS_ values are displayed in Figure 3c. These reveal a steep subthreshold swing (SS) of 140 mV dec^–1^, an on/off current ratio of 10^5^, and reproducibility at various source–drain voltages. All measurements are performed at room temperature, with a transfer curve sweep rate of 0.02 V s^–1^. Notably, the device exhibits minimal hysteresis (∼60 mV; see inset of Figure 3c), especially when considering that phyllosilicate-based dielectrics commonly display higher hysteresis due to impurities^26,36^ or water intercalation.^61^ We attribute the comparatively small hysteresis to the low iron impurity content in our phlogopite crystal and the thermal annealing step preceding the measurements. Furthermore, using phlogopite flakes thinner than ∼10 nm is critical for strong gate control. From the transfer curves, the estimated field-effect mobility is 6 cm^2^ V^–1^ s^–1^ (see Supporting Information for the transconductance curves and complete hysteresis data at various *V*_SD_). The gate leakage of this device is depicted in Figure 3c, corresponding to a maximum normalized leakage of *I*_GS_ = 5 × 10^–5^ A cm^–2^, at an electric field of 2.7 MV cm^–1^.

The density of interface traps (*D*_it_) at the phlogopite/MoS_2_ interface is evaluated via the standard expression:^57^

$$\mathrm{SS}=\ln(10)\frac{k_{B}T}{q}\left(1+\frac{qD_{\mathrm{it}}}{C_{\mathrm{ox}}}\right)$$ 2

where *k*_B_ is the Boltzmann constant, *T* is the temperature, *q* is the elementary charge and *C*_ox_ is the gate dielectric capacitance per unit area (*C*_ox_ = 0.9 μF/cm^2^ for phlogopite with a thickness of 11 nm). We obtain a *D*_it_ of 7.6 × 10^12^ cm^–2^ eV^–1^, comparable to typical values reported for MoS_2_ with conventional oxide dielectrics.^11^ Another key parameter for gate dielectrics in electronics is their equivalent oxide thickness (EOT) compared to SiO_2_, defined as

$$\mathrm{EOT}=t\times \frac{\varepsilon_{{\mathrm{SiO}}_{2}}}{\varepsilon_{r}}$$ 3

where *t* is the thickness of the phlogopite flake, ε_r_ its dielectric constant, and ε_Sio2_ the dielectric constant of SiO_2_ (3.9). The EOT for the sample shown in Figure 3 is therefore 3.7 nm. To reach a desirable EOT of 1 nm, the phlogopite flake needs to have a thickness of 3 nm or less.

To test the buried electrode design further, we fabricated a top-gated FET with monolayer MoS_2_ with a reduced channel length of 3 μm. A gold top-gate is transferred using a PDMS-based van der Waals pickup method. This top-gated device exhibits the same performance metrics (SS = 140 mV dec^–1^) as the device on buried electrodes. Switching to a bilayer MoS_2_ channel with 10 nm of phlogopite on buried electrodes improves the subthreshold slope to ∼100 mV dec^–1^, reduces *D*_it_ to ∼4.2 × 10^12^ cm^–2^ eV^–1^, and lowers hysteresis to ∼30 mV (see the Supporting Information, Figure S7). Such improvements are in line with reports showing that multilayer MoS_2_ FETs often achieve lower SS.^20,57,62^

Finally, we demonstrate the practicality of our buried electrode substrates and phlogopite/MoS_2_ heterostructures by constructing an NMOS logic inverter with an enhancement load (Figure 3d–f). We prepare a substrate containing five buried electrodes, transfer the phlogopite and MoS_2_ flakes, and arrange them so that both transistors share the same phlogopite and monolayer MoS_2_. The MoS_2_ is then laser-patterned into two separate channels (dashed black lines) without damaging the underlying phlogopite.^63^ This “laser trimming” technique allows maximizing the geometry factor (the aspect ratio between the widths of the driver and load transistor), thereby increasing the slope of the inverter’s voltage transfer characteristic. Figure 3e displays the output of the inverter for different *V*_DD_ values; the slight drop in output voltage compared to *V*_DD_ stems from the enhancement load design, an effect that can be mitigated by using a depletion load inverter.^19^ Nonetheless, the inverter maintains a robust voltage gain, as shown in Figure 3f. The flexibility of defining geometry factors is further illustrated in the Supporting Information, where a different laser cut yields a distinct aspect ratio, demonstrating the adaptability of this approach for 2D logic circuits.

### Highly Responsive 2D Photodetectors

In the following, we evaluate the photoresponse characteristics of the MoS_2_/phlogopite phototransistors. The optoelectronic performance of a monolayer MoS_2_ device fabricated on a 29 nm thick phlogopite flake is summarized in Figure 4. An optical micrograph of the device, AFM thickness determination of the phlogopite flake, and a differential reflectance spectrum of monolayer MoS_2_ before transfer are provided in the Supporting Information.

**Figure 4:**
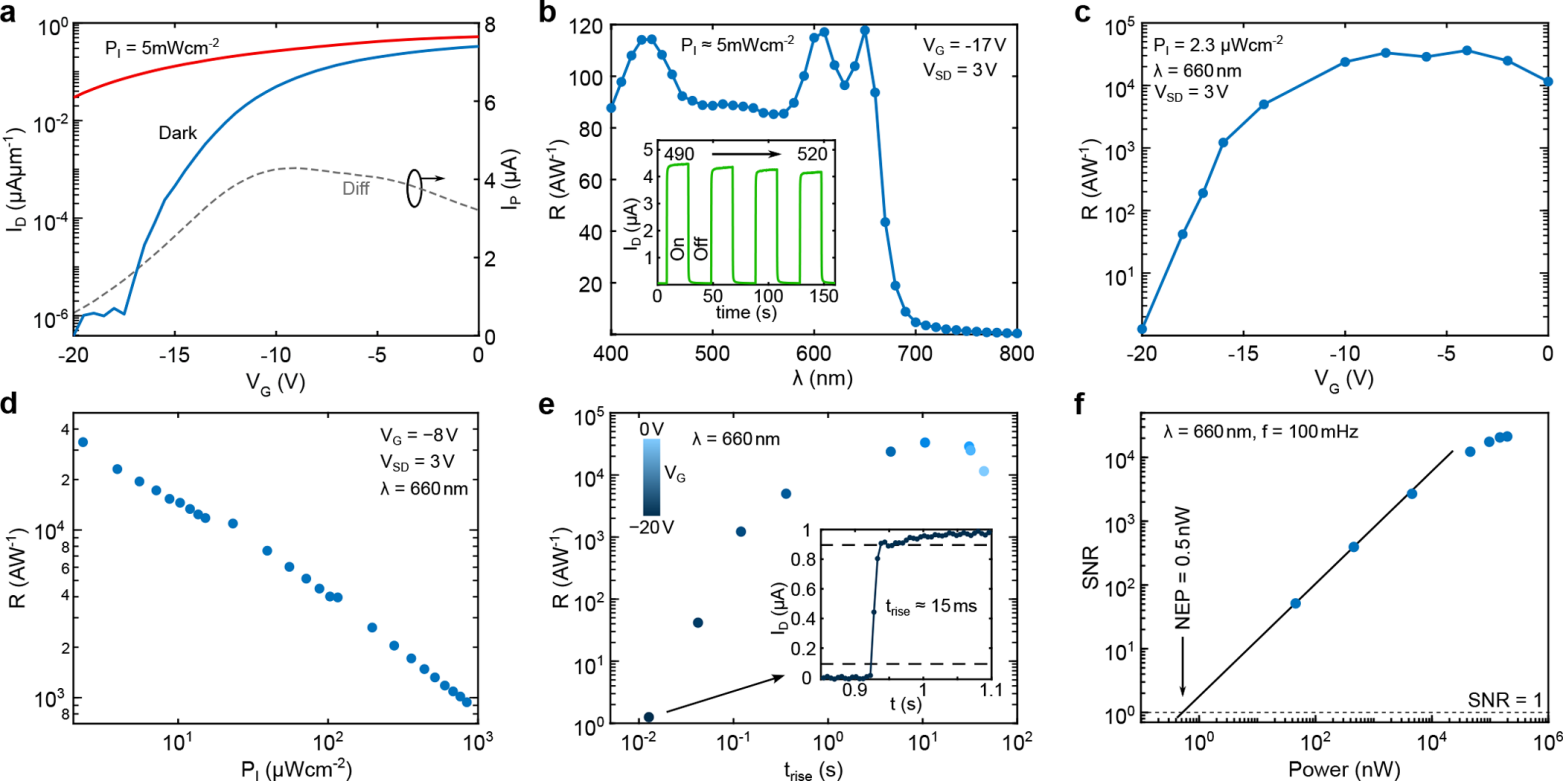
Monolayer MoS_2_ photodetector with phlogopite dielectric. (a) Transfer characteristics of a 1L-MoS_2_ phototransistor before and during illumination under *V*_SD_ = 500 mV and an exposure to a 660 nm LED with *P*_i_ = 5 mW cm^–2^. The panel also plots the difference between the two curves (dashed gray line), identifying the gate voltage with the highest photoresponse (*V*_G_ = −8 V). The phlogopite has a thickness of 30 nm. (b) Wavelength-dependent photoresponsivity under *V*_G_ = −17 V and *V*_SD_ = 3 V. The inset shows selected bias current vs time pulses, from which the responsivity curve is extracted. (c) Gate voltage-dependent responsivity at 660 nm for a fixed illumination power of 2.3 μWcm^–2^. (d) Illumination power-dependent photoresponsivity for a fixed gate (*V*_G_ = −8 V) and source–drain bias (*V*_SD_ = 3 V), with a maximum responsivity of 3.3 × 10^4^ AW^–1^. (e) Response time vs responsivity for different gate bias for a fixed illumination wavelength of 660 nm. Gate bias is depicted by the color intensity. The inset shows the pulse in the *I*–*t* measurement corresponding to the fastest response time of the device (15 ms). (f) Experimental NEP determination for a fixed wavelength and gate bias (*V*_G_ = −17 V). Data points correspond to SNRs extracted by a Fourier transform of the measured photocurrent for decreasing light power from pulsed illumination measurements at *f* = 100 mHz. The intersection between the linear fit with an SNR of 1 gives the NEP for the device from which the detectivity can be calculated.

Figure 4a shows transfer curves of the device before and during illumination (under a source–drain bias of *V*_SD_ = 500 mV and a wavelength of 660 nm) as well as the gate-dependent photocurrent defined as the difference between them. Plotting the difference of the two curves allows identification of the gate bias with the highest photoresponse (*V*_G_ = −8 V). The spectral response of the device in the visible region is illustrated in Figure 4b, extracting the photoresponsivity (*R*) from the measured photocurrent. *R* is defined as the amount of photocurrent generated per optical input power by

$$R=\frac{I_{p}\times A_{L}}{P\times A_{F}}$$ 4

where *I*_p_ is the detected photocurrent (inset of Figure 4b), *A*_L_ is the total illumination area (derived from the spot diameter), *P* is the illumination power, and *A*_F_ is the effective device area. The responsivity plot shows three peaks corresponding to the well-known A (660 nm), B (610 nm), and C (430 nm) excitonic transitions of MoS_2_.

We focus on the device performance at the A exciton wavelength (660 nm) for detailed characterization. Figure 4c shows the gate-dependent R for a fixed light power. As expected, the results follow very closely the shape of the transfer curve difference of Figure 4a: the responsivity is maximized at a gate bias of −8 V and decreases when the phototransistor is turned off. By setting the gate bias for maximal responsivity, we can further analyze the response of the device for decreasing illumination power (Figure 4d). Employing neutral density (ND) filters allows us to further increase R to a value of 3.3 × 10^4^ AW^–1^. This value is exceptionally high compared to previously reported MoS_2_-based phototransistors, even considering devices operating at substantially higher source–drain currents.^7,64,65^

From responsivity measurements, we calculate the photogain (*G*) assuming an external quantum efficiency of unity:

$$G=\frac{R\times h\upsilon }{q}$$ 5

where *h* is Planck’s constant, υ is the frequency of the incident light, and *q* the elemental charge. The calculated maximum G is as high as 6 × 10^4^, typically achievable only in hybrid structures or under significantly higher gate bias.^64,66–68^

Increasing the negative gate voltage reduces the photogating effect, shifting the conduction mechanism of the device to photoconductivity, thereby enabling higher detection speeds.^22^ To illustrate this trade-off, we measure the rise time (*t*_rise_, defined as the time interval for the signal to increase from 10 to 90% of its final value) at different gate biases and correlate it with responsivity (Figure 4e). Responsivity reduction is accompanied by a substantially improved rise time down to 15 ms (fall time *t*_fall_ = 120 ms) at a gate voltage of −20 V, demonstrating dynamic tuning capability.

Naturally, as responsivity increases, the dark current also increases and the response times get longer. On the other hand, a way of identifying the smallest detectable signal of a photodetector is by determining its specific detectivity (D*). We first need to look into the smallest optical power that our device can detect, known as the noise equivalent power (NEP). There are two ways of determining the NEP. First, the NEP can be derived from the power spectral density (PSD) of the photodetector’s noise current and the responsivity, by NEP = PSD/*R*. However, as *R* can depend on the optical power, it is suggested to determine NEP experimentally.^44^ We determine the NEP of our device at *V*_G_ = −17 V by acquiring the power-dependent photocurrent with a modulated optical signal at 100 mHz. By performing a fast Fourier transform (FFT) of our measured current output, we can illustrate the PSD at each illumination power and extract the signal-to-noise ratio^43^ (SNR, Figure 4f). The smallest detectable input power above an SNR of 1 is measured as 0.5 nW. The specific detectivity, considering bandwidth (*f*) and geometry (*A*, effective area of the device), is defined as follows:

$$D^{*}=\frac{\sqrt{A\times f}}{\mathrm{NEP}}$$ 6

Using the experimentally obtained NEP we calculate a specific detectivity of D*_100 mHz_ = 1.5 × 10^8^ J. To compare, we can calculate D* from the dark current PSD (8 × 10^–11^ A Hz^–1/2^; see Supporting Information) and the responsivity (200 AW^–1^, at *V*_G_ = −17 V, see Figure 4c), obtaining 2.16 × 10^8^ J at 100 mHz. The values of the two methods match well, as the responsivity has been acquired at a low power density, close to SNR = 1. Therefore, we can determine the specific detectivity at 1 Hz, which is the preferred value for benchmarking purposes, using the second method. With a PSD of 5 × 10^–12^ A Hz^–1/2^, the detectivity was found to be D*_1 Hz_ = 1.5 × 10^10^ J. At low power densities, the photocurrent behaves according to a power law of *I*_P_ ≈ *P* ^α^, with α = 0.88 (see Table S1 in the Supporting Information for a benchmarking table comparing our phlogopite/MoS_2_ phototransistor with other MoS_2_ photodetectors of various dielectric materials).

To confirm these results, an additional monolayer MoS_2_ phototransistor is fabricated and characterized, showing comparable behavior with a maximal photoresponsivity of 1 × 10^4^ AW^–1^ (see Supporting Information).

To further demonstrate the versatility of phlogopite as a dielectric for 2D optoelectronics, we fabricated a phototransistor using monolayer WS_2_ as the channel material (see Figure S13, Supporting Information). The WS_2_/phlogopite device shows a clear spectral responsivity plot highlighting distinct A (620 nm), B (520 nm), and C (∼430 nm) excitonic peaks, similar to that observed in MoS_2_ devices. The maximum responsivity achieved is 22 AW^–1^ at 617 nm (at a light power of 10 μW cm^–2^), which compares favorably with other WS_2_ phototransistors in the literature^69–71^ and demonstrates the effective photogating in these devices. Compared to our MoS_2_ devices, the WS_2_ device displays lower peak responsivity but maintains high values compared to other studies and clear excitonic features. This difference can be attributed to the higher intrinsic carrier mobility and longer photocarrier lifetimes typically observed in monolayer MoS_2_, as well as potential differences in trap densities and band alignment at the phlogopite interface. Both device types show a low hysteresis and minimal interface trapping. Importantly, the observation of sharp excitonic peaks in WS_2_, together with the high responsivity, highlights the potential of phlogopite as a universal dielectric platform for a range of 2D semiconductors beyond MoS_2_.

## Conclusions

In summary, we demonstrated that naturally occurring phlogopite mica, when exfoliated to the ultrathin limit, functions as a high-quality gate dielectric for monolayer and few-layered TMD transistors and photodetectors. The phlogopite flakes exhibit a wide band gap (∼4.8 eV), a dielectric constant of ∼11, and a high breakdown strength (>10 MV cm^–1^), satisfying key criteria for low-power (opto-)­electronics. By directly integrating phlogopite flakes with MoS_2_, we realized phototransistors with low subthreshold swings (down to 100 mV dec^–1^) that achieve responsivities up to 3.3 × 10^4^ AW^–1^ and detectivities approaching 10^10^ Jones. Moreover, we can effectively tune the photoresponse characteristics of our devices by gate bias. These findings position phlogopite as a sustainable and readily available gate dielectric for advanced 2D electronic and optoelectronic applications, providing new opportunities to push the device performance, reduce power consumption, and enable damage-free integration with various 2D channels.

## Methods

### Materials, Exfoliation, and Transfer

MoS_2_ flakes were mechanically exfoliated from a natural molybdenite mineral (Molly Hill Mine, Quebec, Canada) using Nitto tape (Nitto SPV 224) and phlogopite flakes from its bulk crystal (Madagascar) using Scotch tape (Magic tape by 3M) onto a PDMS substrate (Gel-Film WF 4 × 6.0 mil by Gel-Pack). Phlogopite flakes were transferred to an SiO_2_ (290 nm)/Si (p++) substrate, identified, and stamped to the final substrate using common nail polish transfer.^72^ MoS_2_ flakes were identified on PDMS under an optical microscope^73^ and dry-transferred to the final substrate.^34^

### XPS Characterization

XPS measurements of phlogopite flakes on SiO_2_ (290 nm)/Si substrate were carried out in an ANA chamber (STARDUST machine UHV module)^74^ using a Phoibos 100/150 electron/ion analyzer with a 1D-DL43 2-100 1D delay line detector and XR50 M X-ray source aluminum anode (Al Kα = 1486.7 eV). X-ray was passed through a FOCUS 500/600 monochromator. The analyzer entrance and exit slits were 7 × 20 mm and ‘open’, respectively.

### Buried Electrode Fabrication

Buried electrodes were patterned on an SiO_2_ (290 nm)/Si (p++) wafer by maskless lithography (Microlight3D SmartPrint), a 50 nm etch of the SiO_2_ layer (glass-etching cream Armour Etch) using the photoresist as a mask, and subsequent thermal evaporation of 5 nm Cr and 45 nm Au into the etched trenches.^59,75^

### C-AFM Characterization

A commercial AFM system, from Nanotec, operating under ambient conditions was employed to perform morphological and conductive characterization of the sample. Measurements were acquired in dynamic (topography) and contact modes (C-AFM) using commercial tips from Nanosensors (PPP-FMR) and Rocky Mountain Nanotechnology (25Pt300B), respectively. Current–voltage (*I*–*V*) curves were acquired at different locations in the sample while maintaining a contact force between 200 and 500 nN. During the measurements, a DC bias (ranging from 0 to 9 V) was applied to the tip while the sample was grounded.

### Electrical Characterization

Electrical characterizations were carried out in a home-built measurement setup at room temperature under vacuum conditions (10^–6^ mbar), where the samples can be in situ annealed at 200 °C for 2 h.^76^ For FET measurements, a source-meter unit (Keithley 2450) was used to perform source–drain sweeps, and two programmable benchtop power supplies (Tenma, model 72-2715) are connected in series to perform gate voltage sweeps. For four-probe measurements (double-gated FET (Figure 2d–f), NMOS inverter (Figure 3d–f)), an additional benchtop power supply was used.

### Optoelectronic Characterization

The photoresponse of our devices was tested in the same vacuum chamber as for the electrical measurements. We used multimode fiber-coupled light sources for optical illumination inside the vacuum chamber. The fiber-coupled light is guided through a tube lens system onto our device, resulting in a circular spot of 900 μm diameter with homogeneous power density over the sample.^77,78^ A tunable xenon lamp source (Bentham TLS120Xe) was used to investigate the wavelength-dependent photoresponse. A 660 nm LED source (Thorlabs, MxxxFy series) in combination with ND filters (Thorlabs NEK01S) enabled us to study power-dependent photocurrent generation over a wide range of illumination power.

## Supplementary Material



## Data Availability

The data sets generated during and/or analyzed during the current study are available from the corresponding author on reasonable request.

## Notes

Optical thickness determination of phlogopite flakes (Figure S1); Raman analysis of phlogopite (Figure S2); dielectric constant determination by Schottky emission extraction; Pt–Pt C-AFM curve (Figure S3); frequency-dependent dielectric constant measurement of MIM structures (Figure S4); dielectric breakdown details and material comparison (Figure S5); FET details and hysteresis (Figure S6); bilayer MoS_2_ FET (Figure S7); top-gated MoS_2_ FET (Figure S8); NMOS inverter with different enhancement load (Figure S9); phototransistor device details (Figure S10); NEP determination (Figure S11); phototransistor benchmark comparison table (Table S1); additional MoS_2_ phototransistor (Figure S12); and WS_2_ phototransistor (Figure S13) (PDF)
