# Supplementary material for: Natural Layered Phlogopite Dielectric for Ultrathin Two-Dimensional Optoelectronics
Source: ACS Nano. 2025 Aug 8;19(32):29672–81. doi: 10.1021/acsnano.5c09046 (PMC12369019; doi:10.1021/acsnano.5c09046)
Supplement: Supplementary file 1 [file nn5c09046_si_001.pdf]

Supporting Information:

Natural layered phlogopite dielectric for ultrathin two-dimensional optoelectronics

Thomas Pucher[1], Julia Hernandez-Ruiz[1], Guillermo Tajuelo-Castilla[2], José Ángel Martín-Gago[2], Carmen Munuera[1] and Andres Castellanos-Gomez[1*]*

[1]2D Foundry Research Group. Instituto de Ciencia de Materiales de Madrid (ICMM-CSIC), Madrid, E-28049, Spain.

[2]ESISNA Research Group. Instituto de Ciencia de Materiales de Madrid (ICMM-CSIC), Madrid, E-28049, Spain.

thomas.pucher@csic.es

andres.castellanos@csic.es

Optical thickness determination of phlogopite flakes

The apparent color of phlogopite flakes, which depends on their thickness, can be understood using a multi-layer optical model, shown in previous works [S1,S2]. In this model, incident light strikes a surface—either the substrate or the flake—and then passes through multiple layers until it reaches the silicon substrate (Fig. S1c). At this point, the light retraces its path (assuming normal incidence) and is ultimately detected by the spectrometer. The reflection and transmission of the light within this system can be determined using Fresnel's equations. The reflected intensity for monochromatic light under normal incidence is given by:

$$I(\lambda) = \left| \frac{r_{01}e^{i(\phi_1+\phi_2)} + r_{12}e^{-i(\phi_1-\phi_2)} + r_{23}e^{-i(\phi_1+\phi_2)} + r_{01}r_{12}r_{23}e^{i(\phi_1-\phi_2)}}{e^{i(\phi_1+\phi_2)} + r_{01}r_{12}e^{-i(\phi_1-\phi_2)} + r_{01}r_{23}e^{-i(\phi_1+\phi_2)} + r_{12}r_{23}e^{i(\phi_1-\phi_2)}} \right|^2$$

Where the reflection coefficient is $r_{ij} = (n_i - n_j)/(n_i + n_j)$, the phase shift in the medium is $\phi_i = 2\pi n_i d_i/\lambda$, with its refractive index n_i, its thickness d_i and the wavelength λ. The indices 0,1,2 and 3 are standing for air, phlogopite, SiO_2, and Si, respectively.

The optical contrast can be obtained from

$$C = \frac{I_1 - I_0}{I_1 + I_0}$$

Using this model, in combination with our micro-reflectance setup [S3], we can obtain the flake thicknesses of Figure S1a, optically. The differential reflectance and optical contrast values are plotted in Figure S1c&d. By using AFM to resolve the topography of the flake, we can confirm the accuracy of the optical thickness determination method and determine the index of refraction of phlogopite with 1.45, in agreement with previous work [S4].

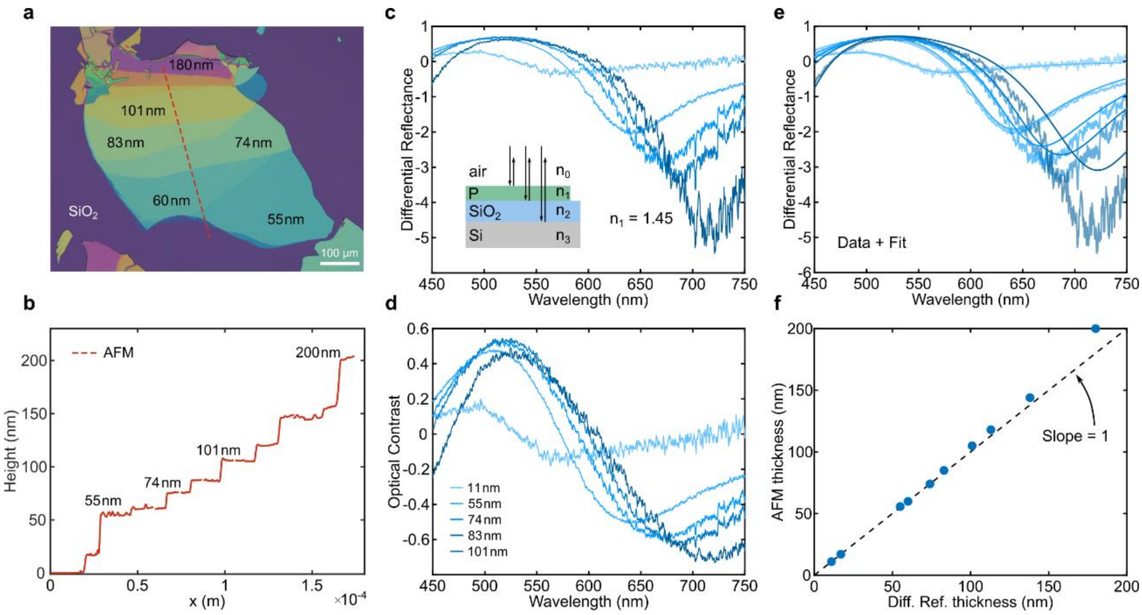

Figure S1. Phlogopite thickness determination. (a) Optical microscopy image of flake with different thicknesses presented in the main text. Indicated thicknesses have been measured optically through micro-reflectance measurements. The red dashed line marks the path of the AFM topography measurements of (b). Phlogopite layer terraces have been measured in individual maps and glued together for demonstration purposes. (c) Differential reflectance curves for selected thicknesses as function of the wavelength. The inset shows an illustration of the different layers involved in the Fresnel law equations, with corresponding refractive index numbers. (d) Optical contrast of the same thicknesses as in (c). The SiO_2 has a thickness of 290 nm. (e) Differential reflectance curves including the corresponding fits. (f) Comparison between thickness values obtained optically and the AFM. The dashed slope of 1 indicates a perfect fit. The optical thickness determination is vary accurate up to around 150 nm of thickness.

Raman analysis of phlogopite

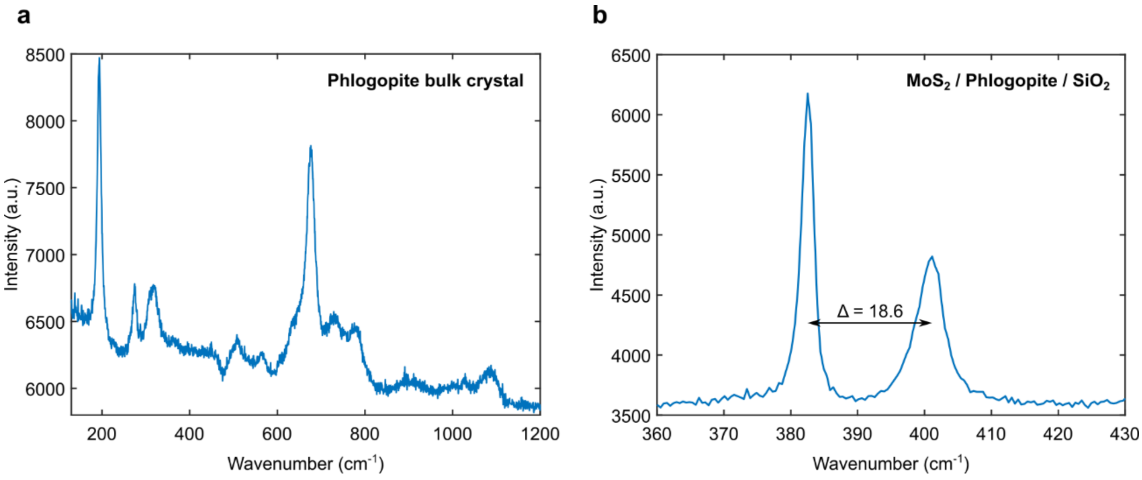

Figure S2. Raman spectroscopy of phlogopite. (a) Raman spectroscopy of bulk phlogopite crystal. (b) Raman spectroscopy of monolayer MoS_2 on top of a phlogopite flake on a SiO_2/Si substrate.

Dielectric constant determination by Schottky emission extraction

Determining the dielectric constant of atomically thin nanosheets can be challenging due to their small lateral dimensions. Fabricating large-scale capacitors with such small samples becomes impractical due to difficulties in ensuring uniformity, reliable contact, and consistent layer thickness over the entire area. By using a conductive AFM tip to apply a voltage and measure the current through the nanosheet, it is possible to characterize the material's electrical properties at a local scale. When an electric field is applied across the nanosheet, the current can be analyzed using the Schottky emission mechanism to extract the dielectric constant of the material. [S26,S27]

Schottky emission or field-assisted thermionic emission, is described by the Richardson-Dushman equation, which states that the current (I) depends on the temperature (T) and the work function (φ):

$$I = AT^2 e^{\left(-\frac{\phi-\Delta\phi}{k_B T}\right)}$$

where $\Delta\phi = \sqrt{\frac{eE}{4\pi\varepsilon_0\varepsilon_r}}$ is the field-dependent reduction in barrier height (A is the Richardson constant, E is the electric field, ε_0 is the vacuum permittivity, k_B is the Boltzmann's constant, and ε_r is the relative permittivity). The electric field across the flake is related to the applied voltage (V) and the flake thickness (d), resulting in a modified Schottky emission equation:

$$I = AT^2 e^{\left(-\frac{\phi-\sqrt{\frac{eV}{4\pi\varepsilon_0\varepsilon_r d}}}{k_B T}\right)}$$

To extract the contribution of the relative permittivity, we linearize this equation taking the natural logarithm to

$$\ln(I) = \ln(AT^2) - \frac{\phi}{k_B T} + \frac{\sqrt{\frac{eV}{4\pi\varepsilon_0\varepsilon_r d}}}{k_B T}$$

Let $C = \sqrt{\frac{e}{4\pi\varepsilon_0 d}}$, the equation becomes

$$\ln(I) = \ln(AT^2) - \frac{\phi}{k_B T} + \frac{C\sqrt{V}}{\sqrt{\varepsilon_r}T}$$

When plotting $\ln(I)$ vs $\sqrt{V}$, the dielectric constant ε_r can be directly extracted from the slope k of the linear region.

$$\varepsilon_r = \left(\frac{C}{k \cdot T}\right)^2$$

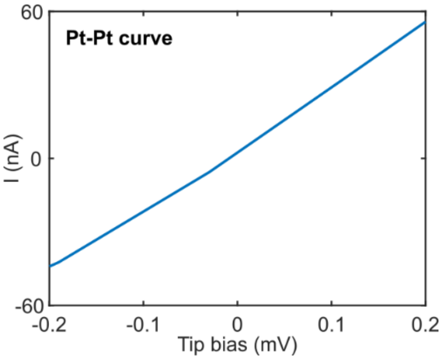

Figure S3. Pt-Pt comparison curve of C-AFM. The platinum tip is positioned on top of the platinum electrode without a flake in between to validate the electrical connection.

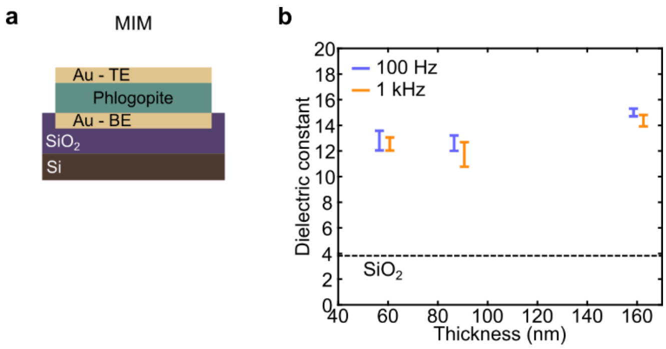

Figure S4. Frequency-dependent dielectric constant measurement of MIM structure. (a) Sketch of the MIM structure. (b) Dielectric constant for various thicknesses determined at 100 Hz and 1 kHz. For comparison the dielectric constant of SiO_2 (3.9) is indicated with a dashed line.

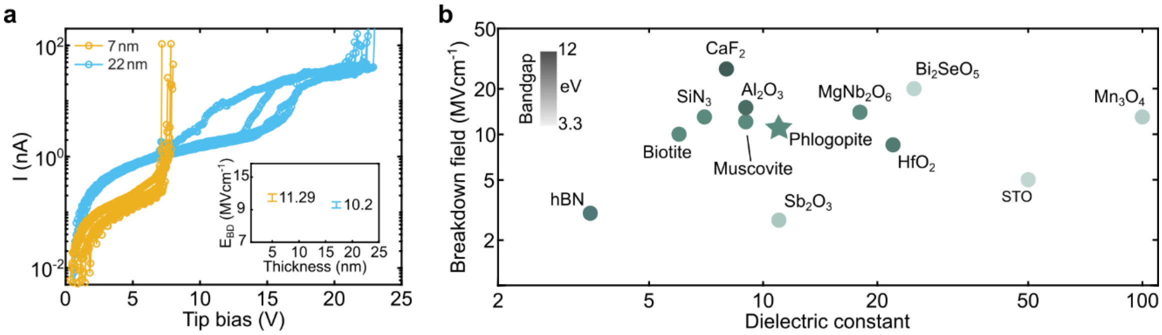

Figure S5. Dielectric breakdown of phlogopite flakes and comparison with other dielectrics. (a) Two flake thicknesses were further investigated under the C-AFM for dielectric breakdown (7 nm and 22 nm). Breakdown fields are indicated in the inset and exceed 1 V/nm. (b) Comparison of different dielectrics suggested for two-dimensional electronics. Bandgap size is indicated by color intensity. Information taken from Refs S5-S25.

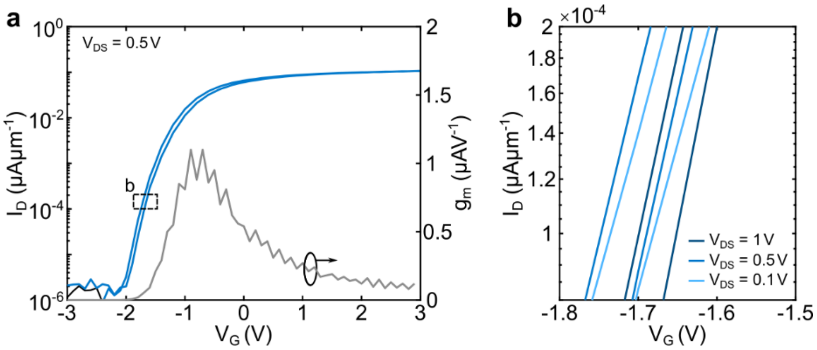

Figure S6. Monolayer MoS2 FET details for the device in the main text. (a) Full transfer curve and extracted transconductance for a V_{DS} of 0.5 V. The maximum transconductance value is taken for field-effect mobility calculations. (b) Magnification of the subthreshold regime of all three transfer curves from the main text, illustrating the consistent hysteresis of ~60 mV even for varying V_{DS}. Sweep rate is 0.02 V/s and all measurements were performed under vacuum conditions (~e-6mbar) at room temperature.

Bilayer MoS_2 FET

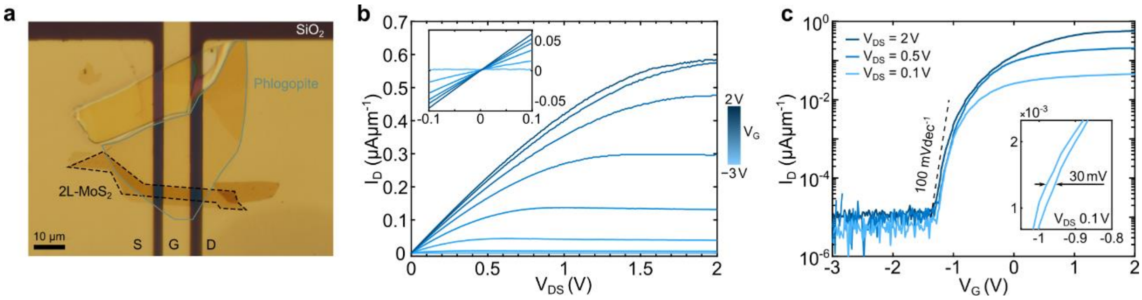

Figure S7. Bilayer MoS_2 FET details. (a) Optical micrograph of the bilayer FET with a phlogopite of 10 nm thickness (EOT = 3.4 nm, W = 4 µm, L = 15 µm). (b) Gate-dependent source-drain current-voltage (IVs) curves showing current saturation at higher V_{DS} and linear behavior at low V_{DS} (inset). (c) FET forward transfer characteristics for different bias voltages. The inset shows the hysteresis of the transfer curve for a source-drain bias of 0.1 V.

Top-gated monolayer MoS_2 FET

We use pre-patterned (buried) electrodes to first transfer single-layer MoS_2 and then thin phlogopite on top. To realise the top-gate gold transfer, we first prepare gold strips of desired size on SiO_2/Si substrate by maskless lithography and thermal Au evaporation (50 nm), without evaporation of a sticking layer. Then we prepare a stamp consisting of a rectangular piece of PDMS (Gel-Film WF 4 × 6.0 mil by Gel-Pack) mounted on a glass slide, overhanging like a cantilever. We bring the PDMS in contact with the gold strip and peel it off as fast as possible to pick up the gold strip. As a final step the gold strip is transferred on top of the MoS_2/phlogopite stack by dry-transfer, acting as a top-gate electrode.

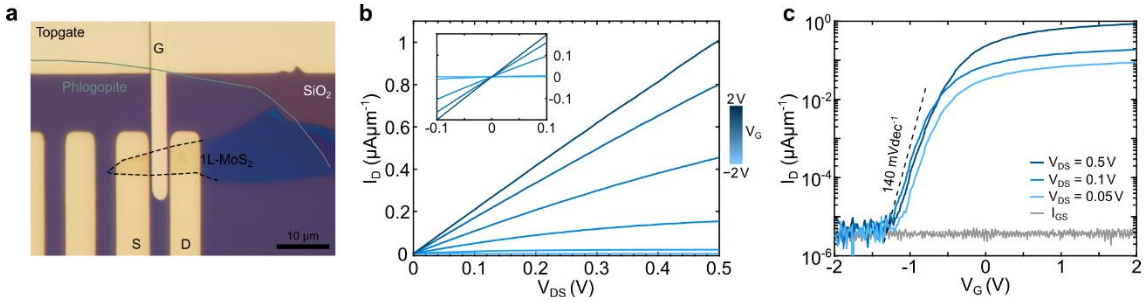

Figure S8. Top-gated monolayer MoS_2 FET details. (a) Optical micrograph of the top-gated FET. The phlogopite has a thickness of 9 nm (EOT = 3 nm, W = 5 µm, L = 3 µm). (b) Gate-dependent source-drain current-voltage (IVs) curves showing current saturation at higher V_{DS} and linear behaviour at low V_{DS} (inset). (c) FET forward transfer characteristics for different bias voltages and gate leakage. The normalized gate leakage has a maximum of $I_{GS} = 1.3\times10^{-4}$ Acm^{-2}, at an electric field of 2.2 $MVcm^{-1}$.

Monolayer MoS_2 inverter with different enhancement load

NMOS inverters usually are categorized in inverters with resistor load, enhancement load or depletion load. Their input-output relationship is subject to the geometry factor between the load and driver transistor. The sharpness of the transition, as well as the minimum output voltage decrease with increasing load resistance. An inverter with enhancement load uses a load transistor instead of a resistor, decreasing its footprint dramatically. The two transistors in the inverter circuit are defined by their threshold voltage and their geometrical aspect ratio K_D/K_L, where K_X is the transistor's width-to-length ratio. As K_D/K_L increases, the input-output relationship of the inverter improves. [S28] Due to the design of our circuit, the lengths of both driver and load transistor is always the same, which means that the aspect ratio is solely defined by the width ratio of the two transistors, which we can define by laser cutting. Illustrating the importance of the aspect ratio, we show the inverter device of the main text after an initial laser cut that only separated the two transistors with one cut (panel a), resulting in an aspect ratio of 1.1. Input-output characteristics and inverter gain are shown below and prove poor device performance. After these measurements load and driver transistor have been laser cut again, to define an aspect ratio of around 4, which corresponds to the results shown in the main text.

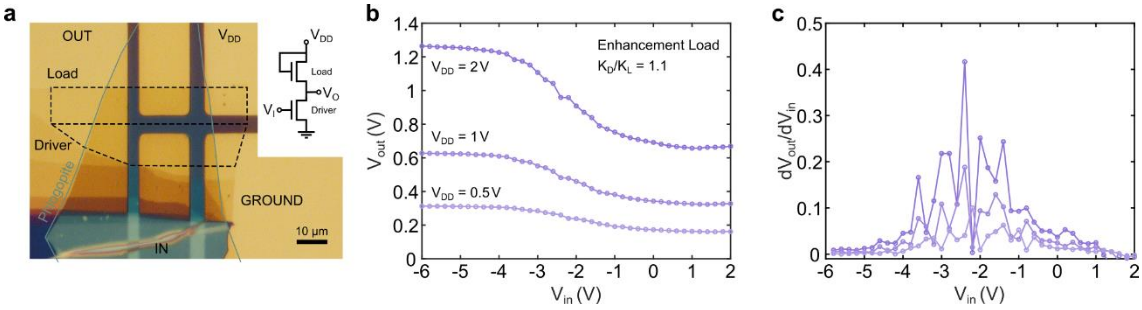

Figure S9. Monolayer MoS2 inverter with different enhancement load. (a) Optical micrograph of single-layer MoS_2 NMOS inverter structure with enhancement load factor of 1.1, before final laser cutting. (b) Inverter characteristics for different V_{DD} voltages. Parasitic effects for this configuration are large. (c) Inverter gain extracted from (b).

Optical microscope image, AFM and differential reflectance of MoS_2 phototransitor

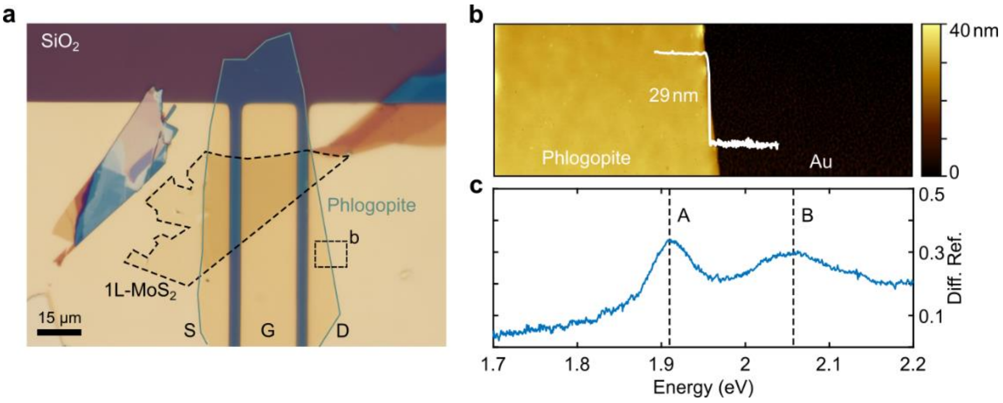

Figure S10. Details of device of main figure 4. (a) Optical micrograph of single-layer MoS_2 phototransistor on a 29 nm thick phlogopite flake. (b) AFM topography of the area marked in (a). (c) Differential reflectance spectroscopy of the MoS_2 flake on PDMS before transfer onto the phlogopite.

Noise equivalent power (NEP) extraction and specific detectivity (D*) calculation

The NEP is defined as the smallest detectable optical power of the photodetector at a certain bandwidth, whereas the detectivity D is the reciprocal of NEP:

$$D = \frac{1}{NEP}$$

The specific detectivity takes bandwidth (f) and geometry factors (A, effective area of the device) into account and is defined by:

$$D^* = \frac{\sqrt{A \cdot f}}{NEP}$$

To determine NEP without overestimating detectivity it is crucial to experimentally measure NEP and therefore the photodetector’s noise. Calculating the noise simply by

$PSD = \sqrt{2qI_{dark}f}$ can lead to a largely underestimated NEP value and therefore overestimated D*.

For example, if we calculate the detectivity of our system with this formula we get a value of $D^*_{1Hz} = 2\times10^{11}$ J, which is one order of magnitude higher than the value from the experimental NEP determination in the main text.

The current spectral density of the device of main Figure 4 is plotted in Figure S9a, including the instrumentation noise floor with lifted probe tips (labelled “open”). We see that 1/f noise (shot noise) is the dominating noise contribution at low frequencies.

To determine the NEP experimentally, we measure photocurrent while modulating the input LED light with a function generator at a frequency of 100 mHz. Doing so for decreasing power allows us to extract signal-to-noise (SNR, difference between the noise floor and the peak amplitude) at the modulation frequency by Fourier transforming the photocurrent measurement.

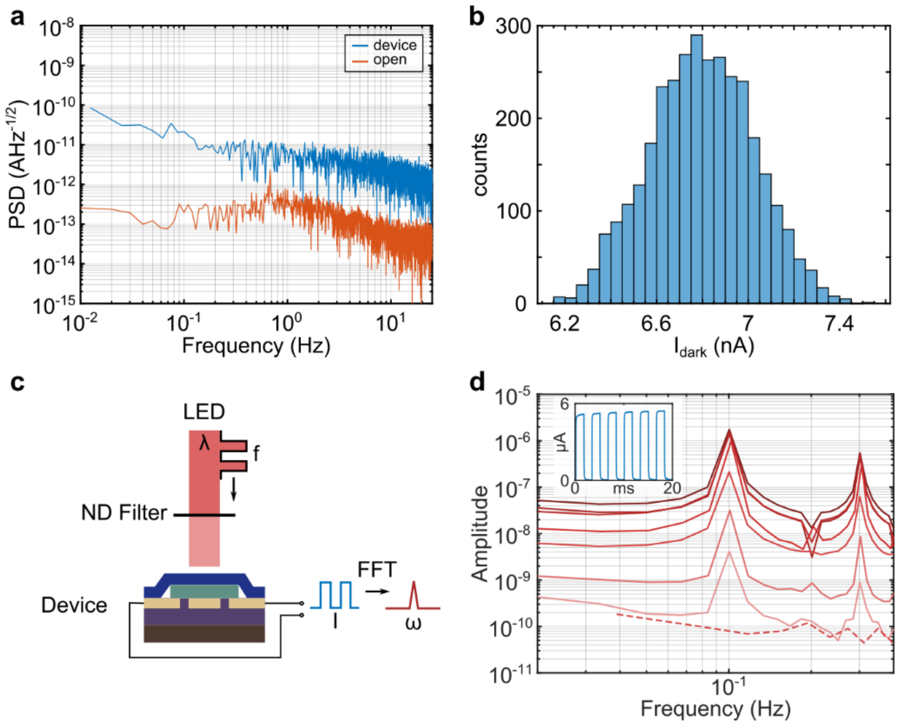

Figure S11. MoS_2 phototransistor noise and NEP determination. (a) Current spectral density of the dark current under a gate bias of V_G = –17 V and V_{SD} = 3 V (device) and with the probe tips lifted (open). (b) Histogram of dark current under the same conditions. (c) Schematic of experimental NEP determination. The light of the LED gets modulated by a function generator with the frequency f (in our case 100 mHz) and passes through neutral density (ND) filters onto the device. The modulated output current (I) is measured with a Keithley source-meter and Fourier transformed to extract the SNR. (d) Fourier transformed current signals (ω) around the modulation frequency. The noise floor (SNR = 1) is indicated by a dashed line. The inset shows the measured output current (I) for a configuration without ND filter. The first harmonic of the square signal appears at 0.3 Hz (3f).

MoS_2 phototransistor benchmarks for various dielectric systems

The following table summarizes figures of merit for phototransistors in literature with the focus on photodetection in the visible red spectrum, comparable to our results.

Material	λ (nm)	R (AW^{-1})	D^* (J)	t rise/fall (ms)	REF
SiO_2	400-600	880	-	4000/9000	S29
SiO_2	532	2200	-	-/55	S30
Al_2O_3	625	$1x10^4$	-	10/0.1	S31
SNO	600	163	3.8×10^{14} *	-	S32
HfO_2	~620	1.3×10^4	-	-	S33
$SiO_2/MoS_2/HfO_2$	635	5×10^4	7.7×10^{11}	7	S34
Si_3N_4	647	~200	-	13000/11000	S35
P(VDF-TrFE)	635	2570	2.2×10^{12} *	10/10	S36
SiO_2	532	0.57	10^{10} *	0.07/0.11	S37
MoO_x	638	10^{-2}	10^{10} *	52/52	S38
Sb_2O_3	671	2×10^4	10^{15} *	60/55	S39
**Phlogopite**	**660**	**3.3×10^4**	**10^{10}**	**15/120**	**This work**

Table S1. MoS_2 phototransistor benchmark comparison. Values for responsivity and detectivity are given as maximum device values, which mostly is not measured at the same gate bias (e. g. photo conduction mechanism).

Note: Specific detectivity values marked with * are not directly measured but calculated using the formula $D^* = R \frac{\sqrt{A}}{\sqrt{2e \cdot I_{dark}}}$ and therefore very likely overestimated, as previously explained.

Additional MoS_2 photodetector device

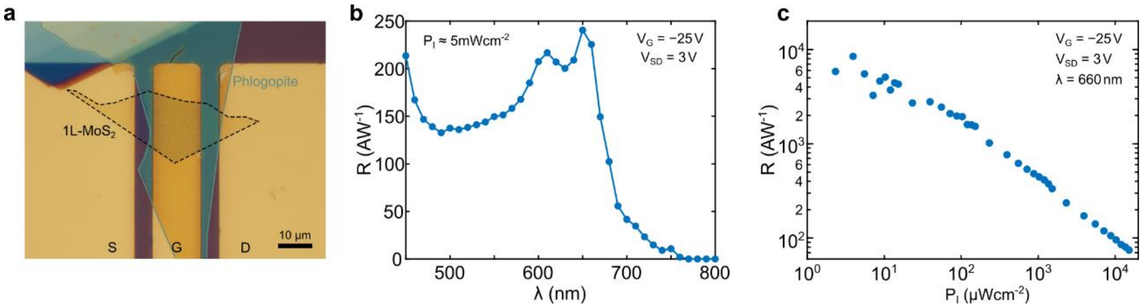

Figure S12. Additional monolayer MoS_2 phototransistor details. a) Optical micrograph of the device. The phlogopite has a thickness of 51 nm. (b) Wavelength-dependent photoresponsivity under V_G = –25 V and V_{SD} = 3 V. (c) Illumination power-dependent photoresponsivity for a fixed gate (V_G = –25 V) and source-drain bias (V_{SD} = 3 V), with a maximum responsivity of 1×10^4 AW^{-1}.

Monolayer WS_2 phototransistor on phlogopite

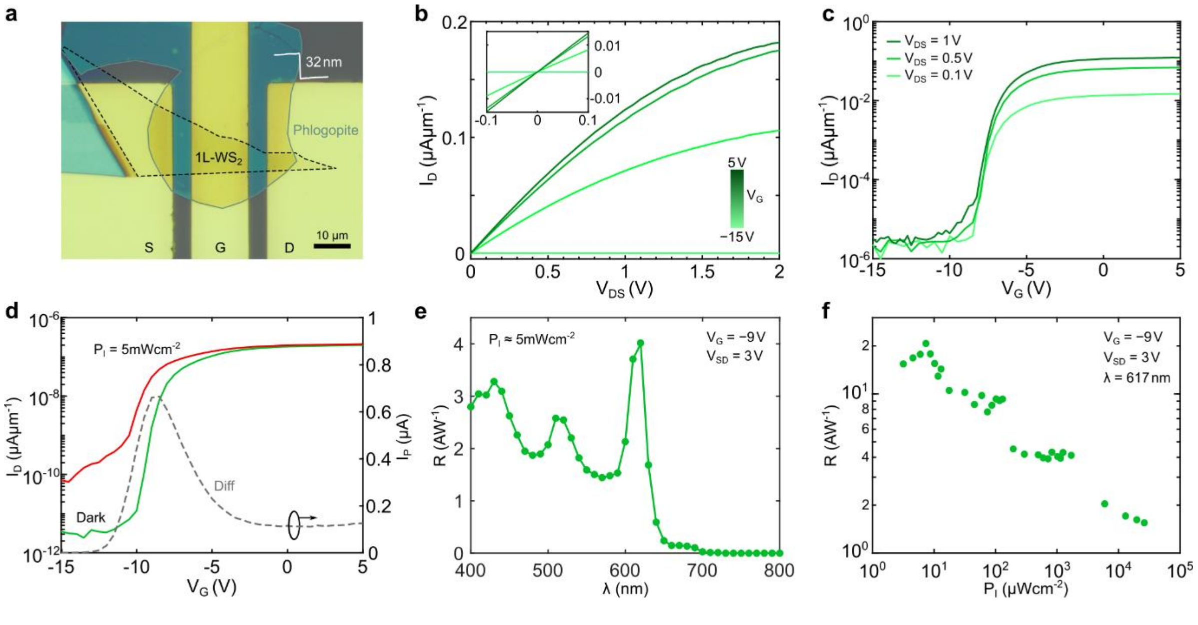

Figure S13. Monolayer WS_2 phototransistor on phlogopite dielectric. (a) Optical micrograph of the WS_2 FET, including an AFM thickness determination. The phlogopite has a thickness of 32 nm. (b) Gate-dependent source-drain current-voltage (IVs) curves. (c) FET forward transfer characteristics for different bias voltages. (d) Pre- and during-illumination transfer characteristics of a 1L-WS_2 phototransistor under V_{SD} = 1 V after exposure with a 617 nm LED with P_i = 5 $mWcm^{-2}$. The panel also plots the difference between the two curves (dashed grey line), identifying the gate voltage with highest photo response (V_G = –9 V). (e) Wavelength-dependent photoresponsivity under V_G = –9 V and V_{SD} = 3 V. (f) Illumination power-dependent photoresponsivity for a fixed gate (V_G = –9 V) and source-drain bias (V_{SD} = 3 V), with a maximum responsivity of 22 AW^{-1}.

SUPPORTING INFORMATION REFERENCES

[S1] Puebla, Sergio, et al. "Apparent colors of 2D materials." Advanced Photonics Research 3.4 (2022): 2100221.

[S2] Li, Hai, et al. "Rapid and reliable thickness identification of two-dimensional nanosheets using optical microscopy." ACS nano 7.11 (2013): 10344-10353.

[S3] Frisenda, Riccardo, et al. "Micro-reflectance and transmittance spectroscopy: a versatile and powerful tool to characterize 2D materials." Journal of Physics D: Applied Physics 50.7 (2017): 074002.

[S4] Haley, Kristine L., et al. "Isolation and characterization of atomically thin mica phyllosilicates." ACS Applied Nano Materials 7.21 (2024): 25233-25240.

[S5] Jang, Sung Kyu, et al. "Synthesis and characterization of hexagonal boron nitride as a gate dielectric." Scientific reports 6.1 (2016): 30449.

[S6] Kim, Ki Kang, et al. "Synthesis and characterization of hexagonal boron nitride film as a dielectric layer for graphene devices." ACS nano 6.10 (2012): 8583-8590.

[S7] Kim, Soo Min, et al. "Synthesis of large-area multilayer hexagonal boron nitride for high material performance." Nature communications 6.1 (2015): 8662.

[S8] Kalita, J. M., and G. Wary. "Estimation of band gap of muscovite mineral using thermoluminescence (TL) analysis." Physica B: Condensed Matter 485 (2016): 53-59.

[S9] Rosenholtz, Joseph L., and Dudley T. Smith. "The dielectric constant of mineral powders." American Mineralogist: Journal of Earth and Planetary Materials 21.2 (1936): 115-120.

[S10] Maruvada, Anirudh, et al. "Dielectric breakdown of 2D muscovite mica." Scientific reports 12.1 (2022): 14076.

[S11] Mahapatra, Preeti Lata, et al. "Synthesis and characterization of biotene: a new 2D natural oxide from biotite." Small 18.27 (2022): 2201667.

[S12] Robinson, D. A. "Measurement of the solid dielectric permittivity of clay minerals and granular samples using a time domain reflectometry immersion method." Vadose Zone Journal 3.2 (2004): 705-713.

[S13] de Oliveira, Raphaela, et al. "Ultrathin natural biotite crystals as a dielectric layer for van der Waals heterostructure applications." Nanotechnology 35.50 (2024): 505703.

[S14] Huang, Jing-Kai, et al. "High-κ perovskite membranes as insulators for two-dimensional transistors." Nature 605.7909 (2022): 262-267.

[S15] Van Benthem, K., C. Elsässer, and R. H. French. "Bulk electronic structure of SrTiO 3: Experiment and theory." *Journal of applied physics* 90.12 (2001): 6156-6164.

[S16] Zhang, Congcong, et al. "Single-crystalline van der Waals layered dielectric with high dielectric constant." Nature materials 22.7 (2023): 832-837.

[S17] Sire, Cédric, et al. "Statistics of electrical breakdown field in HfO2 and SiO2 films from millimeter to nanometer length scales." Applied Physics Letters 91.24 (2007).

[S18] Robertson, John. "High dielectric constant oxides." The European Physical Journal-Applied Physics 28.3 (2004): 265-291.

[S19] Kang, Laegu, et al. "Electrical characteristics of highly reliable ultrathin hafnium oxide gate dielectric." IEEE Electron Device Letters 21.4 (2000): 181-183.

[S20] Lin, H. C., P. D. Ye, and G. D. Wilk. "Leakage current and breakdown electric-field studies on ultrathin atomic-layer-deposited Al2O3 on GaAs." Applied physics letters 87.18 (2005).

[S21] Liu, Kailang, et al. "A wafer-scale van der Waals dielectric made from an inorganic molecular crystal film." Nature Electronics 4.12 (2021): 906-913.

[S22] Ma, T. P. "Making silicon nitride film a viable gate dielectric." IEEE transactions on electron devices 45.3 (1998): 680-690.

[S23] Wen, Chao, et al. "Dielectric properties of ultrathin CaF2 ionic crystals." Advanced Materials 32.34 (2020): 2002525.

[S24] Zhu, Cheng-Yi, et al. "Magnesium niobate as a high-κ gate dielectric for two-dimensional electronics." Nature Electronics 7.12 (2024): 1137-1146.

[S25] Yuan, Jiashuai, et al. "Controllable synthesis of nonlayered high-κ Mn3O4 single-crystal thin films for 2D electronics." Nature Communications 16.1 (2025): 964.

[S26] Zavabeti, Ali, et al. "A liquid metal reaction environment for the room-temperature synthesis of atomically thin metal oxides." Science 358.6361 (2017): 332-335.

[S27] Yang, Kena, et al. "Ultrathin high-κ antimony oxide single crystals." Nature Communications 11.1 (2020): 2502.

[S28] Uyemura, John P. Fundamentals of MOS digital integrated circuits. Addison-Wesley Longman Publishing Co., Inc., 1988.

[S29] Lopez-Sanchez, Oriol, et al. "Ultrasensitive photodetectors based on monolayer MoS2." Nature nanotechnology 8.7 (2013): 497-501.

[S30] Zhang, Wenjing, et al. "High-gain phototransistors based on a CVD MoS2 monolayer." Advanced materials 25.25 (2013): 3456-3461.

[S31] Dodda, Akhil, et al. "Active pixel sensor matrix based on monolayer MoS2 phototransistor array." Nature Materials 21.12 (2022): 1379-1387.

[S32] Li, Siyuan, et al. "Two-dimensional perovskite oxide as a photoactive high-κ gate dielectric." Nature Electronics 7.3 (2024): 216-224.

[S33] Nur, Roda, et al. "High responsivity in MoS2 phototransistors based on charge trapping HfO2 dielectrics." Communications Materials 1.1 (2020): 103.

[S34] Kufer, Dominik, and Gerasimos Konstantatos. "Highly sensitive, encapsulated MoS2 photodetector with gate controllable gain and speed." Nano letters 15.11 (2015): 7307-7313.

[S35] Gonzalez Marin, Juan Francisco, et al. "MoS2 photodetectors integrated with photonic circuits." npj 2D Materials and Applications 3.1 (2019): 14.

[S36] Wang, Xudong, et al. "Ultrasensitive and broadband MoS2 photodetector driven by ferroelectrics." Advanced materials 27.42 (2015): 6575-6581.

[S37] Tsai, Dung-Sheng, et al. "Few-layer MoS2 with high broadband photogain and fast optical switching for use in harsh environments." Acs Nano 7.5 (2013): 3905-3911.

[S38] Im, Healin, et al. "Large-area MoS2-MoOx heterojunction thin-film photodetectors with wide spectral range and enhanced photoresponse." APL Materials 7.6 (2019).

[S39] Ye, Kun, et al. "High-performance broadband photodetectors of heterogeneous 2D inorganic molecular Sb2O3/monolayer MoS2 crystals grown via chemical vapor deposition." Advanced Optical Materials 8.17 (2020): 2000168.
